# A meta-analysis of co-occurrence of non-suicidal self-injury and suicide attempt: Implications for clinical intervention and future diagnosis

**DOI:** 10.3389/fpsyt.2022.976217

**Published:** 2022-08-12

**Authors:** Zhiyu Ye, Fang Xiong, Wentian Li

**Affiliations:** ^1^School of Education Research, China University of Geosciences, Wuhan, China; ^2^Research Center for Psychological and Health Sciences, China University of Geosciences, Wuhan, China; ^3^Wuhan Mental Health Centre, Wuhan, China

**Keywords:** non-suicidal self-injury (NSSI), suicide attempt (SA), co-occurrence, systematic review, meta-analysis

## Abstract

**Background:**

Non-suicidal self-injury (NSSI) and suicide attempt (SA) are risk behaviors that lead to physical injury and even death in individuals, and are a very powerful risk factor when both occur together, with individuals presenting with more severe psychological and behavioral problems. Due to the different demographic characteristics of different study subjects, an overall understanding of the incidence and occurrence of this co-occurrence is lacking to clarify the focus of clinical interventions and future research directions.

**Methods:**

A systematic search was conducted for relevant studies in English and Chinese that reported data on co-occurring non-suicidal self-injury and suicide attempts as of May 2022. The incidence of co-occurrence of non-suicidal self-injury and suicide attempt (NSSI + SA) was calculated using Stata version 16.0 software based on a random-effects model, and the differences in incidence in different populations were compared by subgroups of age group, comorbidity, and time of occurrence. The study was written in strict accordance with PRISMA norms and registration was completed on the PROSPERO platform (CRD42022329095).

**Results:**

A total of 37 studies (139,573 individuals) were included for meta-analysis, and the combined incidence of non-suicidal self-injury and suicide attempt co-occurrence was 9.6%. Among different groups, the prevalence of NSSI + SA was 10, 11, 6, and 26% in adolescents and young adults, adults, the general population, and people with mental illness, respectively, and the co-occurrence of NSSI + SA within 12 months was 17%.

**Conclusion:**

There is a significant group with a history of both non-suicidal self-injury and suicide attempts and presenting with more severe symptoms clinically. Targeted prevention and intervention are urgently needed, but the direction of intervention needs further research on the occurrence trajectory of this co-occurrence.

**Systematic Review Registration:**

http://www.crd.york.ac.uk/PROSPERO/display_record.asp?ID=CRD42022329095, identifier: CRD42022329095.

## Introduction

Non-suicidal self-injury (NSSI) is intentional self-injury to bodily tissue without suicidal intent for socially or culturally unsanctioned purposes (1). A suicide attempt (SA), is a self-destructive act with at least some intent to end one's life (1). Because suicide attempts are one of the strongest predictors of completed suicide in individuals (2), and 10%−15% of patients with prior suicide attempts eventually die by suicide (3), the main focus in previous studies has been on the occurrence of suicide attempts. It is worth noting, however, that NSSI is not a less lethal form of SA, but a coping mechanism to regulate emotions and endure life, SA reflects a desire to end one's life (4).

Although NSSI differs from suicide attempts, the risk of suicide increases over time as habituation to self-harm and physical pain reduces an individual's fear of suicide and death, or as the need for more than what NSSI can provide (5). The literature suggests a strong association between NSSI and SA. Franklin et al. concluded that a history of NSSI is one of the most consistent and powerful predictors of suicidal behavior in studies conducted over a 50-year period (6). Bryan et al. also concluded that a history of NSSI predicted future suicide attempts, and that adolescents with a history of NSSI had a significantly increased risk of suicide attempts in the following 2 years. The risk of suicide attempts was significantly higher among adolescents with a history of NSSI in the following 2 years (7). In a study of community and clinically independent samples, investigations revealed a moderate to high association between NSSI and history of suicide attempts, with the association stronger than many other recognized risk factors for suicide (e.g., depression, anxiety, and borderline personality disorder), and only suicidal ideation was more strongly associated with suicide attempts than NSSI (8). In addition, previous cross-sectional studies of soldiers receiving mental health care (7) and a prospective study of adolescents (9) have found that a history of NSSI is even more predictive of future suicide attempts than a history of previous suicide attempts.

In addition to the exploration of the association between NSSI and suicide attempt as two separate events, many studies have also found considerable overlap between NSSI and SA (10), which can occur not only separately but also and often simultaneously, and are a particularly powerful risk factor (9, 11). Nock et al. (12) found, after clinical interviews with 89 hospitalized adolescent patients, that 70% of adolescents who reported a history of NSSI also reported a history of prior suicide attempts, and this group of patients showed a trend toward more methodologically diverse self-injurious behaviors that require more clinical attention. In other studies, almost 63%−77% of adolescents with psychiatric disorders who reported both NSSI and suicide attempts were also reported (9, 10), a phenomenon that has become increasingly common in clinical practice.

There has been much academic discussion about NSSI and suicide attempt. The controversy has focused on whether NSSI and suicide attempt are distinct concepts or whether they are part of the same set of self-injurious behaviors, and what the connections and differences between the two are (13, 14). Many scholars advocate separating the two concepts in the diagnostic system (15–17), and some studies suggest that this may be more conducive to adequate risk assessment and treatment planning (18).

In light of the available research findings and to encourage further future research, the fifth edition of the Diagnostic and Statistical Manual of Mental Disorders (DSM-5) included the two as two distinct conditions in the Proposed Criteria collection. Through a literature review, data reanalysis, and field trial results, expert consensus identified recommended diagnostic entries, thresholds, and durations (19). However, because there is insufficient evidence to justify the inclusion of these recommendations in Section II of the formal diagnosis of mental disorders, these recommended criteria are not used for clinical application and remain to be further explored. Although there is some variation in previous studies regarding the association between NSSI and suicide attempt, there is an accepted consensus from these studies that there is an underlying intent to die in the act of suicide attempt and not in the act of NSSI, which is the primary difference between the two behaviors in the recommended diagnostic entries in the DSM-5 (20). In the DSM-5, the proposed criterion for non-suicidal self-injury is five or more days of intentional self-injury in the past year in anticipation of physical harm but without suicidal intent. In contrast, the proposed criterion for Suicidal Behavior Disorder associated with a suicide attempt is that the individual has made a suicide attempt within the past 24 months and must not have involved a repeat (at least five times in the past 12 months) self-injurious event.

In summary, previous studies have suggested that we have a significant number of individuals with a history of both NSSI and suicide attempts, and that this population presents with more severe psychological and behavioral problems that require targeted and timely clinical interventions (21). However, due to the different demographic characteristics of the populations investigated in these studies, there is a lack of an overall understanding of the co-occurrence of NSSI and suicide attempt, as well as a review overview of the specific studies, which makes it difficult to identify the focus of clinical interventions and the direction of future research. In addition, because the proposed diagnostic criteria associated with NSSI and suicide attempt in the DSM-5 are incompatible over time, understanding the co-occurrence of the two events may also inform the development of subsequent modifications to the formal diagnosis.

## Method

This study was written in strict accordance with Preferred Reporting Items for Systematic Reviews and Meta-Analyses (PRISMA) guidelines and was registered with PROSPERO (International Prospective Register of Systematic Reviews) and completed the registration with the registration number CRD42022329095.

### Search strategy

This search utilized the literature databases: PubMed, Embase, Cochrane library, CNKI, Wanfang Data, Web of science, PsycINFO (APA PsycNet), ClinicalTrials.gov, and the preprint databases: MedRxiv, ChinaXiv, SSRN, using a combination of search terms (^*^ indicates truncation) including: “non-suicidal self injury,” “non-suicidal self harm,” “NSSI,” “suicide,” “suicide attempt^*^,” “suicide ideation,” a comprehensive review of relevant articles to date was conducted for the period March 1, 2022 to May 1, 2022. This search had no language or country restrictions and comprehensively considered all potential studies that met the criteria.

### Inclusion/exclusion criteria

Two authors (ZY and FX) independently assessed the eligibility of included studies. Studies included in this review had to meet the following inclusion criteria: (1) the study provided cross-sectional data on the prevalence of suicidal behavior and NSSI over 12 months or over a lifetime; (2) the study had a clear definition of suicidal behavior and NSSI. Studies that (1) included only individuals with a history of NSSI or a history of suicide were excluded (22, 23); and (2) the study provided relevant data on the subjects over a 24-h period (24). In case of disagreements, consensus was sought from another author (WL) outside the evaluation.

### Data extraction

Two reviewers (ZY and FX) independently screened the data; disagreements were resolved by discussion. For each eligible study, the following data were extracted: first author, year of publication, country in which the study was conducted, total sample size, mean age of participants, female sex ratio, sample characteristics, number of participants with a history of suicide attempts and NSSI, and time scale of measurement of history of NSSI or suicide attempts. Data on the characteristics of all studies are shown in Table 1.

**Table 1:** Characteristics of the included studies.

**References**	**Study location**	**Sample size**	**Mean age**	**Proportion** ** of female** ** gender**	**Sample**	**SAMPLE characteristics**	**Prevalence of NSSI + SA**	**SA**	**NSSI**
Aboussouan et al. (29)	USA	4,285	40.1	54.7%	Adult	Veterans and non-veterans	0.096	12 month	Lifetime
Ahn et al. (30)	Korea	1,355	23.1	100.0%	Adult	ED patients	0.099	Lifetime	12 month
Andrewes et al. (31)	Australia	107	18.9	83.0%	Youth	BPD patients	0.523	12 month	12 month
Anestis et al. (32)	USA	1,317	21.1	78.8%	Adult	College students	0.089	Lifetime	Lifetime
Asarnow et al. (9)	USA	334	15.9	69.7%	Adolescent	MDD patients	0.137	Lifetime	Lifetime
Baus et al. (33)	Austria	68	27.1	78.0%	Adult	BPD patients	0.294	Lifetime	Lifetime
Bjureberg et al. (18)	Sweden	1,855	15.5	74.6%	Youth	Psychiatric inpatient	0.067	Lifetime	Lifetime
Boxer (34)	USA	476	13.9	47.5%	Adolescent	Psychiatric adolescents	0.308	Lifetime	Lifetime
Brausch and Gutierrez (35)	USA	373	15.0	48.0%	Adolescent	Students	0.040	Lifetime	Lifetime
Brausch et al. (36)	USA	1,232	19.9	71.8%	Adult	College students	0.030	Lifetime	Lifetime
Bryan (7)	USA	422	36.3	27.3%	Adult	Military Personnel and Veterans	0.026	Lifetime	Lifetime
Bryan (7)	USA	176	27.5	13.1%	Adult	Military personnel	0.233	12 month	Lifetime
Chartrand et al. (37)	Canada	264	NA	52.6%	Adult	Psychiatric inpatient	0.254	Lifetime	Lifetime
Cheung et al. (38)	China	2,317	16.4	54.8%	Adolescent	Students	0.047	12 month	12 month
Coppersmith et al. (39)	USA	709	NA	NA	Adult	General population	0.073	Lifetime	Lifetime
Favril (40)	Belgium	1,203	37.7	0.0%	Adult	Male prisoners	0.091	Lifetime	Lifetime
Güney et al. (41)	Turkey	165	36.5	27.3%	Adult	SSD patients	0.248	Lifetime	Lifetime
Jacobson et al. (42)	USA	227	15.1	68.0%	Adolescent	Psychiatric outpatient	0.176	Lifetime	Lifetime
Kearns et al. (43)	USA	357	41.4	19.9%	Adult	Military veterans	0.109	Lifetime	Lifetime
Lear et al. (44)	USA	165	42.9	11.5%	Adult	Veterans	0.061	Lifetime	Lifetime
Liang et al. (45)	China	2,131	13.0	49.1%	Adolescent	Students	0.023	Lifetime	Lifetime
Liu et al. (21)	China	11,831	15.0	49.1%	Adolescent	Students	0.009	Lifetime	Lifetime
Macrynikola et al. (46)	USA	1,712	22.76	81.0%	Adult	College students	0.077	12 month	12 month
Mars et al. (47)	UK	4,850	NA	76.5	Adolescent	General population	0.009	Lifetime	Lifetime
Mork et al. (48)	Norway	251	30.1	42.0%	Adult	Schizophrenia patients	0.143	Lifetime	Lifetime
Muehlenkamp et al. (49)	USA	441	14.9	70.9%	Adolescent	Psychiatric outpatient	0.193	Lifetime	Lifetime
Nielsen et al. (50)	UK	313	19.8	81.0%	Adult	Community Sample	0.121	Lifetime	Lifetime
O'Connor et al. (51)	UK	3,508	NA	49.4%	Adult	General population	0.063	Lifetime	Lifetime
Plener et al. (52)	Germany	665	14.8	57.1%	Adolescent	Students	0.050	Lifetime	Lifetime
Polanco-Roman et al. (53)	USA	352	19.1	74.0%	Adult	College students	0.148	Lifetime	Lifetime
Preyde et al. (54)	Canada	123	15.7	74.4%	Adolescent	Psychiatric inpatient	0.565	Lifetime	Lifetime
Taliaferro et al. (55)	USA	1,635	NA	68.1%were assigned female at birth	Adolescent	Transgender Youth	0.180	Lifetime	12 month
Taliaferro and Muehlenkamp (56)	USA	16,044	NA	64.3%	Adult	College students	0.011	Lifetime	12 month
Taliaferro et al. (57)	USA	61,330	NA	47.2%	Adolescent	Students	0.033	Lifetime	12 month
Tang et al. (58)	China	15,623	15.2	48.5%	Adolescent	Students	0.031	12 month	12 month
Voss et al. (59)	Germany	1,180	17.9	48.3%	Adolescent and young adult	General population	0.066	Lifetime	Lifetime
Wolff et al. (10)	USA	185	15.1	78.1%	Adolescent	Psychiatric inpatient	0.427	Lifetime	Lifetime

### Data analysis

All statistical analyses were performed using Stata version 16.0, and the results for the incidence of non-suicidal self-injury and suicide attempts (NSSI +S A) were combined, with 95% confidence intervals used for all results.

Heterogeneity was assessed by the *I*^2^ statistic, *p*-values of the chi-square test for heterogeneity, and visual inspection of forest plots. Higher values of the *I*^2^ statistic indicate higher levels of heterogeneity, with 25% being low heterogeneity, 50% being moderate heterogeneity, and 75% being high heterogeneity (25). Given the diversity of participants between studies, the heterogeneity of the combined results was expected to be high, and the combined analysis of incidence was calculated using a random effects model.

To explore possible sources of heterogeneity and to understand the incidence of NSSI + SA among participants with different characteristics so that to determine the focus population for intervention, subgroup analyses were performed for outcomes with sufficient inclusion in the study, with subgroup analyses including: age of the sample (Adolescents & Youths, Adults); psychiatric co-morbidity (yes, no). Time scale of NSSI + SA (12-month NSSI + SA, other); geographic location, and economic and social development status (developed countries, developing countries).

Sensitivity analysis was performed by removing three or more studies with a high risk of bias to determine the effect of risk of bias on the results. Publication bias was assessed by looking at contour-enhanced funnel plots (26) and performing Egger's intercept test (27). A *p*-value less than or equal to 0.05 was considered a statistically significant value of publication bias, and the trim and fill method (28) was used to adjust for possible bias.

## Results

**Figure 1:**
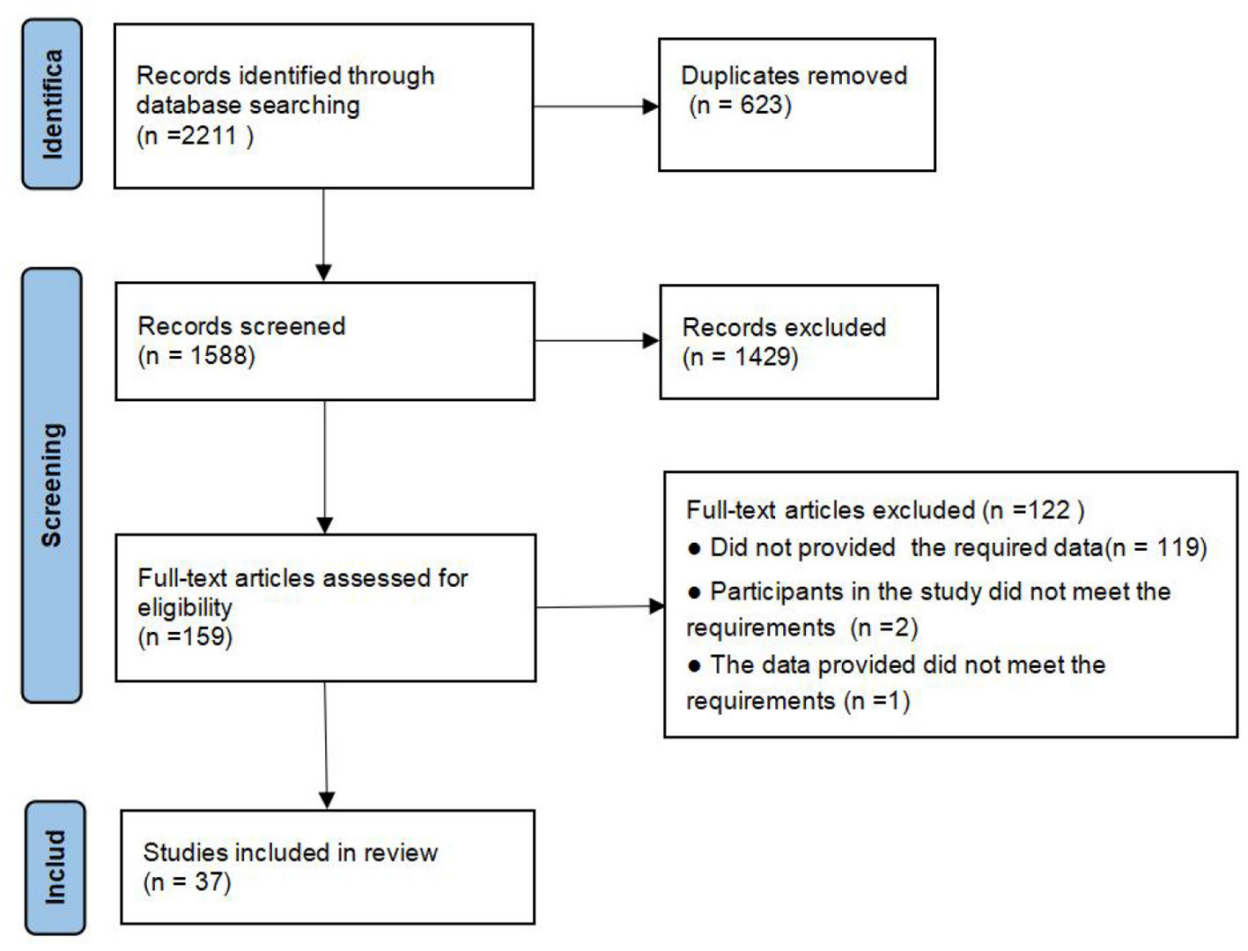
Flow chart of PRISMA study.

After an extensive literature search of the database, a total of 2,211 potentially relevant citations were collected. After eliminating duplicates, the remaining 1,588 studies were screened for titles and abstracts, 1,429 studies that did not meet the inclusion criteria were excluded, and finally 159 studies were comprehensively reviewed, 37 studies that met the criteria were used in this meta-analysis at last. The detailed process of paper selection and reasons for exclusion are shown in Figure 1.

### Study characteristics

In the 37 studies included in the meta-analysis, there were 139,573 participants from 13 countries. The sample size of these studies ranged from *N* = 68 to 61,330, and the mean age of participants ranged from 13.0 to 42.9 years.

In terms of age group, 18 studies had adolescents and youth as participants, 15 of which had adolescents as participants, two studies had youth as participants, and one study had both adolescents and youth as participants. The remaining 19 studies investigated adult individuals. In terms of co-morbidity, participants in 13 studies were outpatients/inpatients with different types of psychiatric disorders, and participants in 24 studies were in the general population. Regarding the time scales of NSSI and SA measured, seven studies measured participants' history of NSSI within 12 months, 29 studies measured participants' lifetime history of NSSI, and only one study was measured with reference to the definition of NSSI in the DSM5; six studies measured participants' history of suicide attempts within 12 months, and 31 studies measured participants' lifetime suicide attempt history; only four studies measured participants' history of NSSI + SA within 12 months. Detailed study characteristics are shown in Table 1.

### Total co-occurrence of NSSI + SA

**Figure 2:**
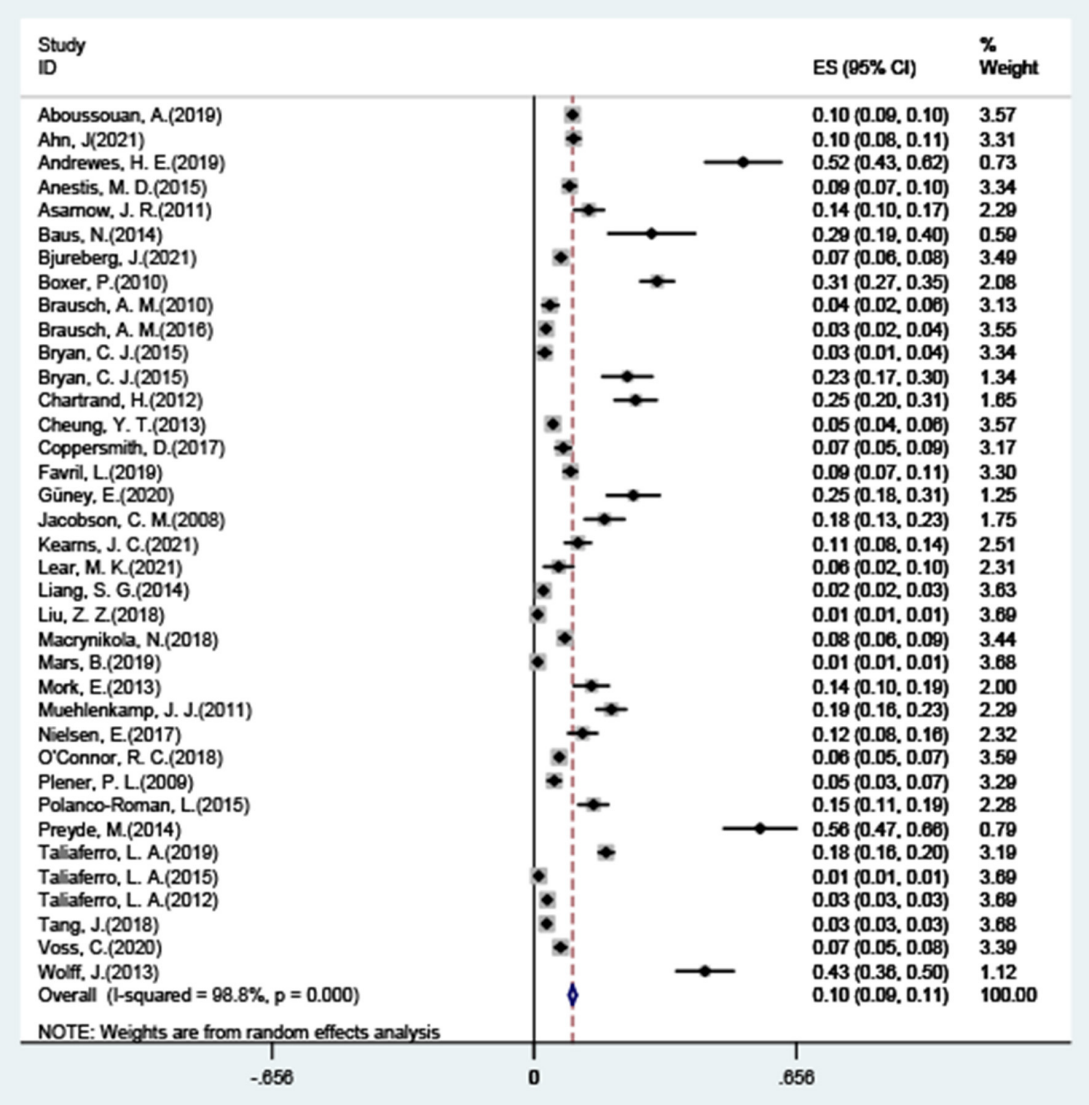
Co-occurrence of NSSI + SA.

**Figure 3:**
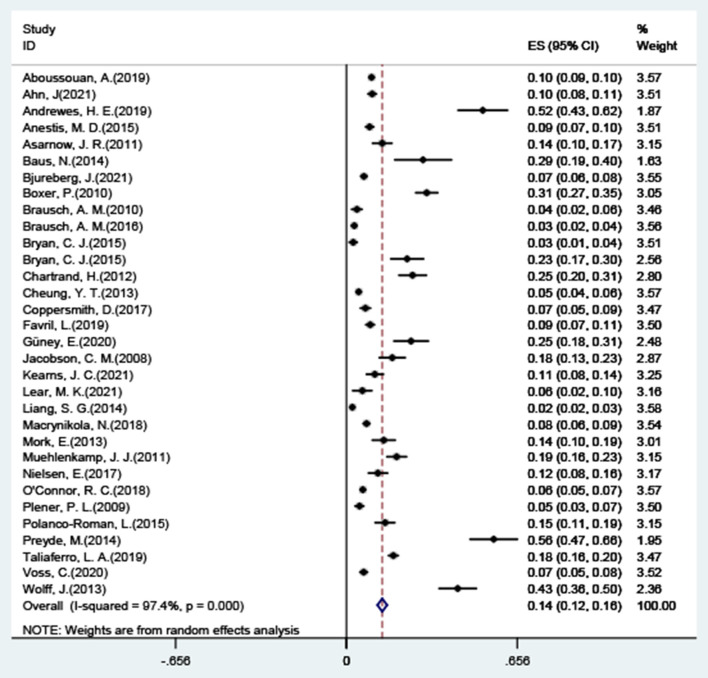
Co-occurrence of NSSI + SA (excluding outliers).

The results of the combined analysis showed that the overall co-occurrence of NSSI and suicide attempts was 9.6% (95% CI: 0.09–0.11), and the forest plot results are shown in Figure 2, with significant high levels of heterogeneity across all included studies (*I*^2^ = 98.8%, 95% CI: 98.60–98.91; *p* < 0.001). After excluding five studies with significant outlier outcomes (21, 47, 56–58), the incidence was 13.7% (95% CI: 0.12–0.16; *I*^2^ = 97.4%, 95% CI: 96.92–97.82; *p* < 0.001), the specific results are shown in Figure 3.

Egger's test suggested some possible publication bias (intercept = 7.96, 95% CI: 5.52–10.40, *t* = 6.62, *df* = 36, *p* < 0.001), and the estimate of the total incidence was 3.3% (95% CI: 0.02–0.04) after correction for publication bias by the cut-and-patch method.

### Subgroup analysis

By combining the different subgroups, the results showed that the overall co-occurrence of NSSI + SA was slightly lower in adolescents and young adults (10%, 95% CI: 0.08–0.11; *I*^2^ = 98.5%, 95% CI: 98.81–99.16) than in adults (11%, 95% CI: 0.08–0.13; *I*^2^ = 99.0%, 95% CI: 98.1–98.76). The total co-occurrence of NSSI + SA was significantly lower in the general population (6%, 95% CI: 0.05–0.07; *I*^2^ = 98.8%, 95% CI: 98.58–98.96) than in patients with psychiatric disorders (26%, 95% CI: 0.20–0.32; *I*^2^ = 98.8%, 95% CI: 96.66–98.08) within 12 months. The overall concurrent incidence of NSSI events with SA events within 12 months was 17% (95% CI: 0.15–0.18; *I*^2^ = 100.0%, 95% CI: 98.08–99.04).

In the general population, the overall incidence of NSSI + SA was lower in adolescents and young adults (5%, 95% CI: 0.03–0.06; *I*^2^ = 99.0%, 95% CI: 98.80–99.24) than in adults (8%, 95% CI: 0.06–0.11; *I*^2^ = 98.6%, 95% CI: 98.26–98.80). Among patients with psychiatric disorders, the total incidence of NSSI + SA was higher in adolescents and young adults (29%, 95% CI: 0.19–0.40; *I*^2^ = 98.3%, 95% CI: 97.69–98.77) than in adults (20%, 95% CI: 0.12–0.28; *I*^2^ = 93.0%, 95% CI:86.54–96.32).

In a broader societal context, continent-based subgroup analyses revealed NSSI + SA rates of 12% (95% CI: 0.11–0.14; *I*^2^ = 98.9%, 95% CI:98.73-99.08) in North America, 5% (95% CI: 0.03-0.07; *I*^2^ = 98.7%, 95% CI:98.17-99.08) in Asia, and 8% (95% CI: 0.05–0.11; *I*^2^ = 98.2%, 95% CI:97.61-98.69) in Europe. In addition, the total NSSI + SA rate was higher in developed countries (11%, 95% CI:0.10–0.12; *I*^2^ = 98.8%, 95% CI:98.58-98.92) than in developing countries (4%, 95% CI:0.02-0.5; *I*^2^ = 98.6%, 95% CI:97.91-99.05).

## Discussion

The present study found a concurrent incidence of 9.6% for NSSI and suicide attempt, this suggests that there is a larger group of people presenting with both NSSI and suicide attempts, which requires urgent attention and effective intervention. Given that studies have shown a significant co-occurrence of NSSI and suicidal behavior in adolescents and young adults (12, 60, 61), we analyzed adolescents and young adults as a subgroup in comparison to the adult sample. The results showed that the co-occurrence of NSSI + SA was 10% in adolescents and youth and 11% in adults in the overall sample, with small differences between subgroups. However, after also considering comorbidity, the results showed that the co-occurrence of NSSI + SA was slightly lower in adolescents and youth than in adults in the general population, while the co-occurrence of NSSI + SA was obvious higher in adolescents and youth than in adults in patients with psychiatric disorders. Based on this, it is necessary to take into account the age and comorbidity background of individuals in clinical prevention and intervention of different groups. For the general population, more attention should be paid to adult population about the NSSI + SA related risk factors, while for psychiatric patients, adolescents and young adults are high-risk groups of NSSI + SA. Moreover, NSSI + SA rates vary between countries with different economic and social development status and continents to which they belong, and need to be viewed in the context of different cultural backgrounds.

One way of thinking about why NSSI and suicide attempts co-occur has been suggested by Joiner in his interpersonal psychological theory of suicide (5), where he argues that a person must have both the desire to commit suicide and the ability to inflict fatal self-injury in order to complete a suicide or make a medically serious suicide attempt. In contrast, there are usually two different interpretations of the order in which the two behaviors occur (62). The first conception holds that individuals first develop suicidal desires and then engage in NSSI, and as the repeated behaviors accustom individuals to fear of self-directed violence and gain tolerance for physical pain, this increases their ability to make serious suicidal injuries and thus suicide attempts. That is, NSSI emerges as a mediating step in suicide attempts. The second conception, on the other hand, suggests that individuals first engage in NSSI without suicidal intent, and then gradually develop the desire to commit suicide through the habituation and acquisition of competence described above, which in turn leads to the emergence of suicide attempts. It is not surprising to find that both sets of explanations assume that NSSI occurs prior to a suicide attempt, although this trajectory assumption was unproven at the time.

In fact, in our literature screening, we found two studies that have now investigated the co-occurrence trajectory of NSSI and suicide attempt events by interview. In a study of a non-clinical sample, Bryan et al. found both cases of NSSI occurring first and cases of SA occurring first. NSSI was approximately 10 times more likely to occur prior to the first suicide attempt than the reverse, and the transition time was approximately 4.5 years for individuals who first engaged in NSSI followed by a suicide attempt. They found that a more common pathway for the two behaviors to occur together was to begin with suicidal ideation, followed by NSSI as an intervention step, and finally a suicide attempt, a developmental trajectory that accounted for 37% of all suicide attempt cases (7). This finding is broadly consistent with Joiner's view that NSSI is used as a mediating step in SA. Whereas in the study by Kearns et al. no cases were found in which suicide attempts appeared before NSSI, the results of that study showed that the transition time from NSSI to suicide attempts was about 4.25 years in psychiatric inpatients (43) and about 7.5 years in the community population (44). We can find some differences between the two studies in terms of the time of transition and the group of subjects investigated. It is also important to note that the subjects in both studies were discharged/non-discharged adult military personnel, so further research needs to be conducted as to whether the results can be applied to a broader group.

Regardless of who preceded or followed these two behaviors, it has been clinically found that individuals with a history of both NSSI and a history of suicide attempts are of greater concern. Previous studies have shown that individuals who report the presence of both a history of NSSI and a history of SA will present with more severe symptoms clinically than individuals who report only a history of NSSI or SA (11, 14, 41), and they have been found to have higher rates of psychiatric diagnoses, more severe psychological or behavioral problems (21), more severe psychiatric symptoms and psychosocial disorders, and higher rates of suicide ideation (40). This can be testified to some extent with the results of our study, where we found a 6% co-occurrence of NSSI and suicide attempts in the general population, and up to 26% in patients with psychiatric disorders, where clinical intervention is indeed imminent. As previous studies on the co-occurrence of the two suggest, NSSI may mark a prodromal period of increased risk for suicide attempts, and effective clinical interventions for patients who self-injure may prevent or halt the transition to suicide attempts, thereby reducing the rate of suicide attempts (7). Therefore, it is recommended that clinicians can regularly screen and assess all NSSI to determine if the motivation for the behavior has changed and if the original purpose is still being achieved, thus assessing whether there is an increased risk of suicide attempts (63). In addition, some scholars suggest that when intervening in NSSI, clinicians should avoid making individuals stop self-harming immediately, as this may eliminate their only coping mechanism, cause them to increase distress and turn to more lethal self-harming methods or suicide attempts (56).

In the current DSM-5, the time frame of the diagnosis is five or more days of intentional self-injury in the past year for non-suicidal self-injury, and a suicide attempt within the past 24 months and must not have involved a repeat self-injurious event for Suicidal Behavior Disorder. Most of the existing studies measure the occurrence of NSSI and suicide attempts over a 12-month or lifetime period in individuals, focusing on whether they have a history of the relevant behaviors and less on when and how many times the relevant behaviors occurred. Although the lack of data clearly defining the occurrence of NSSI for more than 5 days in a year does not fully correspond to the criteria in the DSM-5, we know from meta-analysis that the co-occurrence of both NSSI and suicide attempts in a 12-month period is 17%, and this proportion of individuals who co-occur in both behaviors is not negligible in clinical practice. Thus regardless, in the future development of the DSM, if the inclusion of non-suicidal self-injury and suicidal behavior disorder in the formal diagnostic system is to be considered, the definition of these groups with a history of both NSSI and suicide attempts within a year needs to be looked at carefully. Perhaps a new diagnosis that accommodates both behaviors will be needed, or perhaps the incompatible parts of the existing proposed entry will need to be adjusted. In conclusion, the inclusion of a mental disorder diagnosis is a decision that needs to be considered comprehensively, and this decision should take the actual situation into full consideration in order to formulate the corresponding diagnostic threshold indicators.

In addition to the above findings and reflections, there are some limitations in this study. First, because of the great geographical and cultural variability of the subject groups investigated in different studies, and the data on NSSI and SA reported in different studies were not consistent in terms of definition (time of occurrence, number of times, etc.), so the meta-analysis section showed a large heterogeneity of the combined, at the same time the lack of data on specific characteristics of the subjects made it difficult to eliminate the effect of heterogeneity through subgroup analysis. Second, the present study only conducted subgroup analysis on age, psychiatric comorbidity, NSSI + SA time scale, geographical location, economic and social development status, but did not conduct subgroup discussion on gender, emotional temperament type, mainly because the included study did not provide participants' data of NSSI + SA history at these two feature points. However, given that previous studies have shown that gender is an important factor and the relationship between NSSI and suicide attempts may be stronger in females than in males (7), and studies have found significant gender differences in individuals' NSSI + SA histories, at the same time, the measurement of affective temperament type was independently and closely related to the related clinical outcome (64). Therefore, in the future relevant research, further exploration of individuals with different genders and different emotional temperament types can be considered. In addition, considering that there may be some time transition between the occurrence of both NSSI and suicide attempts, it cannot be ruled out that some participants who have not yet attempted suicide in the present but will eventually do so at some point in the future when cross-sectional surveys are conducted, so more prospective studies are needed to control for this limitation.Last but not least, some scholars have found that subjects at risk for suicidal behavior or NSSI usually approached suicide through searching information and news regarding self-harm and suicidal behaviors on Internet (65). Therefore, in future studies, researchers can further verify whether individuals with a history of NSSI experience the process of Internet information search in the process of turning to suicide attempt and are affected accordingly, so as to confirm the direction of clinical intervention in this dimension.

## Data availability statement

The original contributions presented in the study are included in the article/supplementary material, further inquiries can be directed to the corresponding author/s.

## Author contributions

ZY contributed to the conceptualization, methodology, software, investigation, formal analysis, data curation, and writing—original draft. FX contributed to the investigation, formal analysis, and writing—review and editing. WL contributed to the conceptualization, supervision, validation, and data curation. All authors approved the final version of publication.

## Conflict of interest

The authors declare that the research was conducted in the absence of any commercial or financial relationships that could be construed as a potential conflict of interest.

## Publisher's note

All claims expressed in this article are solely those of the authors and do not necessarily represent those of their affiliated organizations, or those of the publisher, the editors and the reviewers. Any product that may be evaluated in this article, or claim that may be made by its manufacturer, is not guaranteed or endorsed by the publisher.
